# Inhibition of Key Enzymes Linked to Type 2 Diabetes and Sodium Nitroprusside Induced Lipid Peroxidation in Rats’ Pancreas by Phenolic Extracts of Avocado Pear Leaves and Fruit

**Published:** 2014-09

**Authors:** Ganiyu Oboh, Adelusi Temitope Isaac, Ayodele Jacobson Akinyemi, Richard Akinlolu Ajani

**Affiliations:** 1 Functional Foods and Nutraceuticals Unit, Department of Biochemistry, Federal University of Technology, Akure, Nigeria, P.M.B. 704, Akure, 340001, Nigeria;; 2 Department of Biochemistry, Ladoke Akintola University of Technology, Oyo State, Nigeria, P.M.B 4000, Nigeria;; 3 Department of Biochemistry, Afe Babalola University, Ado-Ekiti, Nigeria, P.M.B. 5454, Nigeria

**Keywords:** Type 2 diabetes, lipid peroxidation, Persea americana, α-glucosidase, α-amylase

## Abstract

*Persea americana* fruit and leaves had been known in folk medicine for their anti-diabetic prowess. Therefore, this study sought to investigate the inhibitory effect of phenolic extract from avocado pear (*Persea americana*) leaves and fruits on some key enzymes linked to type 2 diabetes (α-amylase and α-glucosidase); and sodium nitroprusside (SNP) induced lipid peroxidation in rats’ pancreas *in vitro*. The phenolic extracts of *Persea americana* fruit and leaves were extracted using methanol and 1M HCl (1:1 v/v). Thereafter, their inhibitory effects on sodium nitroprusside induced lipid peroxidation and key enzymes linked to type 2 diabetes (α-amylase and α-glucosidase) were determined *in vitro*. The result revealed that the leaves had fruit of avocado pear inhibit both α-amylase and α-glucosidase activities in a dose dependent manner. However, the Peel had the highest α-amylase inhibitory activity while the leaf had the highest α-glucosidase inhibitory activity as revealed by their IC_50_ value. Furthermore, incubation of the rat pancreas in the presence of 5 mM SNP caused an increase in the malondialdehyde (MDA) content in the tissue, however, introduction of the phenolic extracts inhibited MDA produced in a dose dependent manner. The additive and/or synergistic action of major phenolic compounds such as syringic acid, eugenol, vnillic acid, isoeugenol, guaiacol, kaemferol, catechin, ρ-hydroxybenzoic acid, ferulic acid, apigenin, naringenin, epigallocatechin, epicatechin, lupeol and epigallocatechin-3-O-gallate in avocado pear using gas chromatography (GC) could have contributed to the observed medicinal properties of the plant. Therefore, inhibition of some key enzymes linked to type 2 diabetes and prevention of oxidative stress in the pancreas could be some of the possible mechanism by which they exert their anti-diabetic properties

## INTRODUCTION

Improper glucose metabolism affects cellular balance of carbohydrate and lipid metabolism during onset and development of type 2 diabetes (1). Deregulation of these metabolisms lead to postprandial hyperglycemia and later cause noninsulin-dependent type 2 diabetes (2). Diabetes mellitus (DM) is a chronic disease caused by inherited or acquired deficiency in insulin secretion and by decreased sensitivity of the organs to secreted insulin (3). Type 2 diabetes and non-alcoholic fatty liver disease (NAFLD) have insulin resistance as one of their symptoms (4) and visceral fat has been reported from various studies as a major risk factor for insulin resistance and type 2 diabetes (5). The impairment of insulin responsiveness had been reported to be initiated by lipids and their derivatives. This promotes the instability of plaque in the arterial wall and also contributes to inflammatory liver – a condition strictly linked to obesity and fatty liver disease (6).

The end products of α-amylase (EC 3.2.1.1) hydrolysis of starch are maltose, maltotriose, a-dextrins and some glucose. These products are hydrolyzed into their component monosaccharides by enzymes present on the brush border of the small intestinal cells generally called α-glucosidases which are maltase, sucrase, isomaltase and lactase (7-9). This causes a sudden rise in blood glucose level (hyperglycemia) which is a serious complication associated with type 2 diabetes.

One of the most potent adopting therapeutic approaches in the management of hyperglycemia is the reduction of gastrointestinal glucose production and absorption through the inhibition of carbohydrate digesting enzymes such as α-amylase and α-glucosidase. Inhibition of these two carbohydrate hydrolyzing enzymes can significantly decrease the postprandial increase of blood glucose after a mixed carbohydrate diet and therefore can be an important strategy in the management of blood glucose (10).

It has been proved experimentally and clinically that oxidative stress is involved in the development and progression of diabetes mellitus which is due to the deleterious invasion of free radicals or impaired antioxidant defense mechanisms (11, 12). Increased lipid peroxidation and insulin resistance had been linked with abnormal high levels of free radicals and the simultaneous decline of antioxidant defense mechanisms (11).

Drugs called oral anti-diabetics used for the management of type 2 diabetes are potent α-glucosidase inhibitors and they work by preventing carbohydrate digestion thereby reducing the impact of carbohydrate hydrolysis on blood sugar. Glucosidase inhibitors have been available for a long time as prescription medicines, but their use is infrequent as a treatment for diabetes. Acarbose - a common glucosidase inhibitor is used scarcely because of its low efficacy in decreasing glycemic levels. Acarbose elicit unpleasant side effects and are not well accepted by both patients and physicians (13). On the other hand, it has been shown that some plant preparations containing glucosidase inhibitors are devoid of these side effects (touchi, green tea) (14).

Practitioners of folk medicine have been treating diabetes for centuries and claim to have effective treatments for the disease as well as treatment for eradicating other symptoms arising from it (15). Substances that prevent dietary starch from been absorbed by the body are present in amylase inhibitors (starch blockers) (16). α-Amylase inhibitor proteins A-1 and B-2 have been isolated and characterized from cocoyam (*Colocasia esculenta*). These proteins may have potential in the prevention/management of obesity and diabetes (17).

Secondary metabolites like phenolic compounds are synthesised by plants due to their adaptation to biotic and abiotic stress conditions such as infection, water stress and cold stress (18). Edible plants like vegetables and fruits containing polyphenolic compounds had been suggested to have protective role against various forms of diseases (19). Plants have always been an excellent source of drugs and many of the currently existing drugs have been derived directly or indirectly from them (20). Previous study revealed the presence of α-glucosidase inhibitors in seaweeds (21). The antidiabetic and antioxidant properties of polyphenols have been established (22). Some anti-diabetic medicinal plants used which include *Amaranthus cruentus* and *Moringa oleifera* have been shown to have significant high phenolic content. The methanolic extract of the investigated samples showed promising levels of α-amylase (10–45 per cent) and α-glucosidase (13–80 per cent) inhibition activities (23).

The cytotoxicity of sodium nitroprusside (a pro-oxidant and antihypertensive drug) has been reported to involve the release of cyanide and/or nitric oxide (NO) and that NO, is involved in the pathophysiology disorders such as seizure disorders, trauma and stroke and other degenerative diseases (24, 25). Increment in the body’s antioxidant status is one of the ways to combat degenerative diseases and this could be achieved by the higher consumption of vegetables and fruits. Researchers had proved that foods from plant usually contain natural antioxidants that can scavenge free radicals (26).

Avocado pear (*Persea americana*) fruit is not sweet but fatty, flavored, and has a smooth, creamy texture. Avocado fruit in many countries such as Mexico, Brazil, South Africa and India are frequently used for milkshakes and occasionally added to ice-cream. Furthermore, the leaves and seed of *P.americana* have been reported to possess anti-diabetic properties (27).

The focus of this experiment was to establish the usage of *Persea americana* in folklore for the management of diabetes and also to investigate the possible mechanisms of action of avocado pear (*Persea americana*) in the management/prevention of type 2 diabetes mellitus through the investigation of their free radical scavenging ability, inhibition of α-amylase, α-glucosidase and malondialdehyde produced by sodium nitroprusside induced lipid peroxidation in rats’ pancreas.

## MATERIALS AND METHODS

### Materials

The Avocado pear (*Persea americana)* leaves and fruits were obtained from a farm land at Ijoka, Akure, Ondo state and the authentication was carried out at the Department of Crop, Soil and Pest Management, Federal University of Technology, Akure, Nigeria. All chemicals and reagents used in this study were of analytical grade and glass-distilled water was used. A JENWAY UV-visible spectrophotometer (Model 6305; Jenway, Barlo world Scientific, Dunmow, United Kingdom) was used to measure absorbance.

### Phenolic extract preparation

The leaves and fruits were rinsed with distilled water after which the peel, flesh and seed were chopped into pieces and air-dried before milling into a fine powdery form. Then, the total phenolics of the samples were extracted with 1M HCL and methanol solution (1:1 v/v) and filtered (filter paper Whatman number 2) under vacuum. The filtrate was then evaporated using a rotary evaporator under vacuum at 45°C. Then, the phenolic extracts were reconstituted in distilled water (1:100 w/v) and stored in the refrigerator for subsequent analysis.

### α-Amylase inhibition assay

Appropriate dilutions (0-200 µL) of the extracts and 500 µL of 0.02M sodium phosphate buffer (pH 6.9 with 0.006M NaCl) containing porcine pancreatic α-amylase (EC 3.2.1.1) (0.5 mg/mL) were incubated at 25°C for 10 min. Then, 500 µL of 1% starch solution in 0.02 M sodium phosphate buffer. Thereafter, the reaction mixture was incubated at 25°C for 10 min and 1.0 mL of dinitrosalicylic acid (DNSA) was added. Then the reaction was stopped by incubating in a boiling water bath for 5 min and later cooled to room temperature. The reaction mixture was then diluted by adding 10 mL of distilled water, and absorbance was measured at 540 nm in a spectrophotometer (28). The reference sample included all other reagents and the enzyme with the exception of the test sample. The α-amylase inhibitory activity was expressed as percentage inhibition.

$$\text{Inhibition}\;(\%)\;=\;[({\text{Abs}}_{\text{ref}}-{\text{Abs}}_{\text{sample}})/{\text{Abs}}_{\text{ref}}]\;\times \;100$$

where Abs_ref_ = absorbance of the reference; Abs_sample_ = absorbance of the test sample.

### α-Glucosidase inhibition assay

Appropriate dilutions of the extracts (0-200 µL) and 100 µL of α-glucosidase (EC 3.2.1.20) (0.5 mg/mL) in 0.1M phosphate buffer (pH 6.9) solution were incubated at 25°C for 10 min. Then, 50 µL of 5 mM *p*-nitrophenyl-α-D-glucopyranoside in 0.1 M phosphate buffer (pH 6.9) solution was added. The mixtures were incubated at 25°C for 5 min, before reading the absorbance at 450 nm in the spectrophotometer (29). The reference sample included all other reagents and the enzyme with the exception of the test sample. The α-glucosidase inhibitory activity was expressed as percentage inhibition.

$$\text{Inhibition}\;(\%)\;=\;[({\text{Abs}}_{\text{ref}}-{\text{Abs}}_{\text{sample}})/{\text{Abs}}_{\text{ref}}]\;\times \;100$$

### Lipid peroxidation assay

**Preparation of Tissue Homogenates.** The rats were sacrificed under mild diethyl ether anaesthesia and the pancreas was rapidly isolated and placed on ice and weighed. This tissue was subsequently homogenized in cold saline (1:5 w/v) with about 10-up-and–down strokes at approximately 1200 rev/min in a Teflon glass homogenizer. The homogenate was centrifuged for 10 min at 3000 × *g* to yield a pellet that was discarded, and a low-speed supernatant (SI) was kept for lipid peroxidation assay (30).

**Lipid peroxidation and thiobarbituric acid reactions.** The lipid peroxidation assay was carried out using the modified method of Ohkawa *et al.*, (1979) (31). Briefly 100 µL of SI fraction mixed with a reaction mixture containing 30 µl of 0.1M pH 7.4 Tris-HCl buffer, extract (0–100 µL) and 5 µM sodium nitroprusside (SNP) was prepared. The volume was made up to 300 µl by distilled water before incubation at 37°C for 3 hours. The colour reaction was developed by adding 300 µl 8.1% SDS (sodium dodecyl sulphate) to the reaction mixture containing SI; this was subsequently followed by the addition of 600 µl of acetic acid/HCl (pH 3.4) mixture and 600 µl 0.8% TBA (thiobarbituric acid). This mixture was incubated at 100°C for 1 hour. TBARS (thiobarbituric acid reactive species) produced were measured at 532 nm and expressed as malondialdehyde (MDA) equivalent.

### 2, 2’-Azino-bis (3-ethylbenzthiazoline-6-sulphonic acid) (ABTS^*^) scavenging ability

The ABTS^*^ scavenging ability of the extracts was determined according to the method described by Re *et al.* (1999) (32). The ABTS^*^ was generated by reacting an (7 mmol/l) ABTS^*^ aqueous solution with K_2_S_2_O_8_ (Potassium peroxosulfate) (2.45 mmol/l, final concentration) in the dark for 16hr and adjusting the absorbance at 734 nm to 0.700 with ethanol. 0.2 ml of appropriate dilution of the extract was then added to 2.0 mL ABTS^*^ solution and the absorbance were taken at 734 nm after 15 minutes. The TROLOX equivalent antioxidant capacity (TEAC) was subsequently calculated.

### Nitric oxide (NO) scavenging ability

Nitric oxide scavenging assay was performed using gries reagent method reported by Sufanta *et al.* (2006) (33). Briefly, 1 mL each of various concentration of the extract (0.1–0.4 mg/mL), 0.3mL of sodium nitroprusside (5 mM) were added. The text tubes were then incubated at 25°C for 150 minutes. 0.5 ml of gries reagent (equal volume of 1% sulphanilamide on 5% autophosphoric acid and 0.01% naphthlethylmediamine in distilled water used after 12 hrs of preparation) was added. The absorbance was measured at 546 nm.

The percentage inhibition of OD was calculated by using the formula:

% increase in absorbance =[(Blank OD-Test OD)/Blank OD] ×100%

where OD = Optical density.

### Characterization of phenolic constituents using gas chromatography (GC) analysis

The qualitative-quantitative analysis of the phenolic compounds of the samples was carried out using the method reported by Kelly *et al.* (1994) (34). The phenolic compounds were extracts from each sample as described by Kelly *et al.* (1994) (34). After extraction, the purified phenolic extracts (1 µL: 10:1 split) were analyzed for composition by comparison with phenolic standards (Aldrich Chemical Co., Milkwaukee, W1) and a chromatography with standards on a Hewlett-Packard 6890 gas chromatography (Hewlett-packard Corp., Palo Alto, CA) equipped with a derivatized, nonpacked injection liner, a Rtx-5MS (5% DIPHENTL-95% dimethyl polysiloxane) capillary column (30 m length, 0.25 mm column id., 0.25 µm film thickness), and detected with a flame ionization detector (FID). The following conditions were employed PA separation; injection temperature, 230°C; temperature ramp, 80°C for 5 min then ramped to 250°C at 30°C/min; and a detector temperature of 320°C.

### Determination of IC_50_ Values

IC_50_ (extract concentration causing 50% enzyme inhibition and 50% neutralization of reactive oxygen species) values for assays were calculated using nonlinear regression analysis after plotting percentage inhibition of enzymes and reactive oxygen species against extract concentration in mg/mL.

### Data Analysis

Readings were taking in triplicates, pooled and expressed as mean ± standard deviation. For data analysis, the software SPSS was used. A two way analysis of variance (ANOVA) was used to treat difference between means while Duncan multiple test was used as for the post hoc analysis. Significance was accepted at *P*≤0.05.

## RESULTS

**Figure 1:**
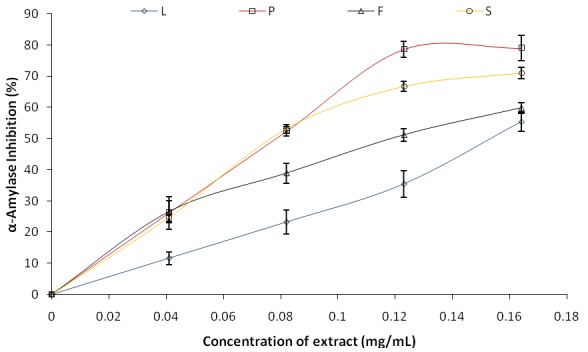
α-Amylase inhibition activity of *Persea americana* leaves and fruit parts extract.

**Figure 2:**
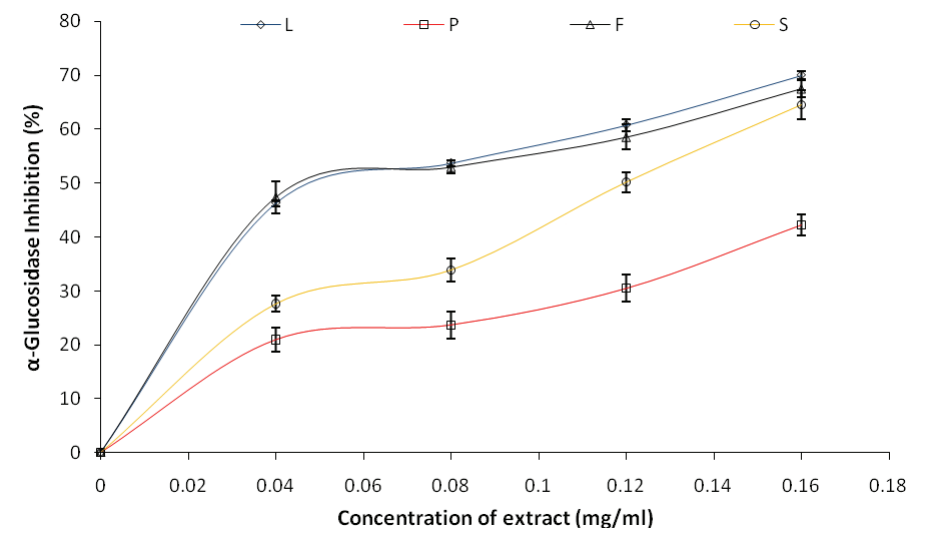
α-Glucosidase inhibition activity of *Persea americana* leaves and fruit parts extract.

The inhibitory effect of Avocado (*Persea americana*) pear leaves and fruit parts on both α-amylase and α-glucosidase are presented in Fig. 1 and Fig. 2 respectively. The IC_50_ (extract concentration that will inhibit 50% enzyme activity) was also estimated as shown in Table 1 below. The result revealed that all the extracts inhibited α-amylase in a dose-dependent manner (in the range of 0–0.164 mg/mL), however, as revealed by the IC_50_ values (Table 1), phenolic extract from the peel of avocado pear (IC_50_=0.057 mg/mL) had the highest inhibitory effect on α-amylase activity while the leaf (IC_50_=0.219 mg/mL) had the least. Also, the ability of the phenolic extracts from avocado pear (*Persea americana*) fruit parts and leaves to inhibit α-glucosidase activity *in vitro* was investigated and the result is presented in Figure 2. All the extracts exhibited a dose-dependent enzyme inhibitory activity in the range of 0–0.164 mg/mL, however, the leaf (IC_50_=0.067 mg/mL) exhibited the highest inhibitory effect while the peel (IC_50_=0.241 mg/mL) had the least when taking into account the IC_50_ values of the phenolic extracts (Table 1).

**Table 1:** IC_50_ (extract concentration causing 50% inhibitory effect) values of α-amylase and α-glucosidase inhibitory activities by phenolic extract of avocado pear (*Persea americana*) leaves and fruit parts

Sample	IC50 (mg/mL) α-Amylase inhibitory activity	IC50 (mg/mL) α-Glucosidase inhibitory activity

Leaf	0.219^a^ ± 0.012	0.067^a^ ± 0.001
Peel	0.057^b^ ± 0.001	0.241^b^ ± 0.011
Flesh	0.079^c^ ± 0.028	0.079^c^ ± 0.006
Seed	0.077^c^ ± 0.002	0.116^d^ ± 0.009

Valuesrepresent Mean ± Standard deviation of triplicate readings. Values with the same superscript along the column are not significantly (*P*<0.05) different.

**Figure 3:**
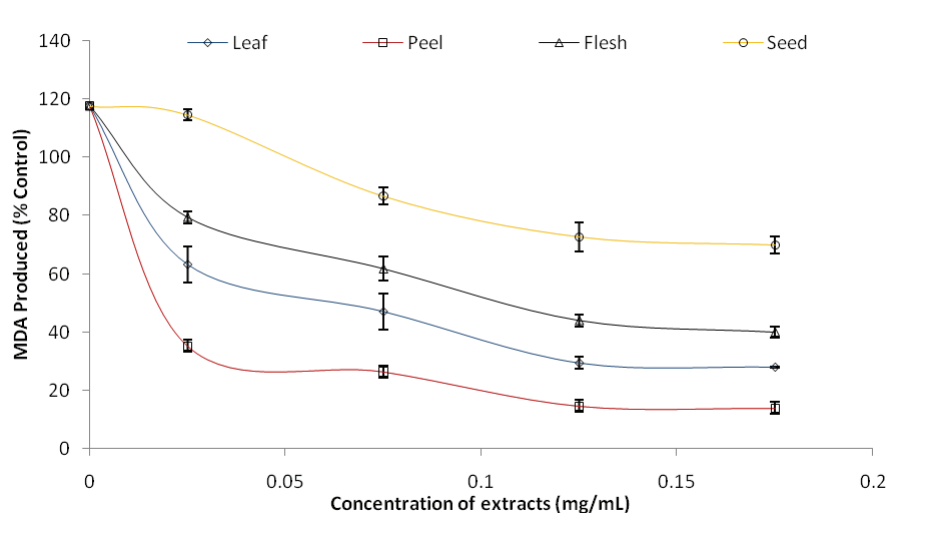
Inhibition of MDA produced by SNP induced lipid peroxidation by *Persea americana* leaves and fruit parts phenolic extract.

Incubation of rat’s pancreas tissue homogenates in presence of sodium nitroprusside (SNP) also caused a significant increase (*P*<0.05) in the rat pancreas malondialdehyde (MDA) content as shown in Figure 3; however, all the extracts inhibited malondialdehyde production content in the tissue in a dose-dependent manner (0–0.175 mg/mL). Phenolic extract from the peel of avocado pear (IC_50_=0.038 mg/mL) had the highest inhibitory effect on SNP induced lipid peroxidation in the pancreas while the seed (IC_50_=0.139 mg/mL) had the least (Table 2).

**Table 2:** IC_50_ (extract concentration causing 50% inhibitory effect) values of NO radical scavenging ability and MDA inhibitory activity by phenolic extract of avocado pear (*Persea americana*) leaves and fruit parts

Sample	IC50 (mg/mL) NO Scavenging activity	IC50 (mg/mL) MDA inhibitory activity

Leaf	0.096^a^ ± 0.008	0.061^a^ ± 0.004
Peel	0.075^b^ ± 0.004	0.038^b^ ± 0.001
Flesh	0.102^c^ ± 0.010	0.085^c^ ± 0.006
Seed	0.090^a^ ± 0.003	0.139^d^ ± 0.005

Values represent Mean ± Standard deviation of triplicate readings. Values with the same superscript along the column are not significantly (*P*<0.05) different.

**Figure 4:**
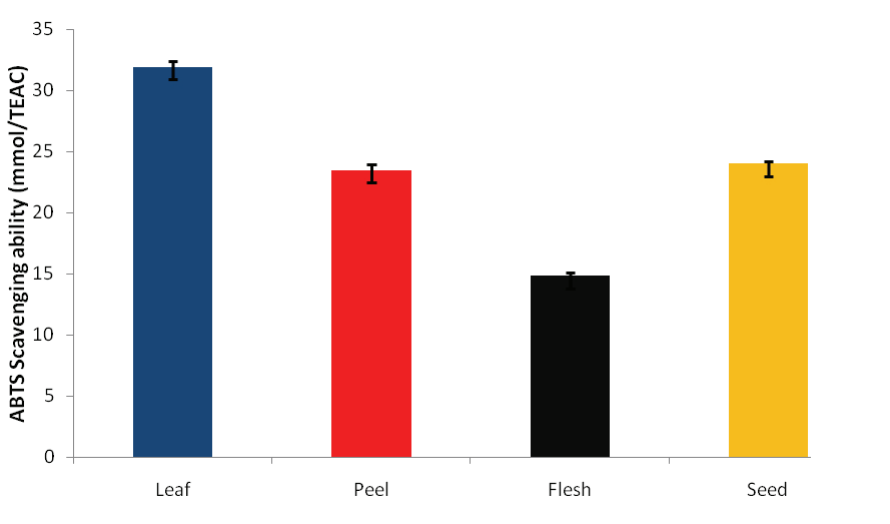
ABTS^*^ radical scavenging ability of phenolic extracts of leaves and fruit parts of *Persea americana*.

The ABTS^*^ scavenging ability presented as trolox equivalent antioxidant capacity is presented in Figure 4. The results revealed that all the extracts scavenged ABTS (2, 2’-azino-bis (3-ethylbenzthiazoline-6-sulphonic acid) free radical, however, the phenolic extract of the leaf (31.88 mmol. TEAC/100g) had the highest ABTS^*^ scavenging ability while the flesh (14.79 mmol. TEAC/100g) had the least.

**Figure 5:**
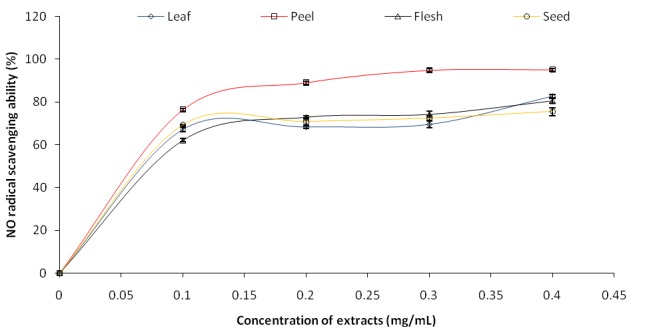
Nitric oxide (NO) radical scavenging ability of phenolic extracts of leaves and fruit parts of avocado (*Persea americana*) pear.

The nitric oxide (NO) radical scavenging ability of the phenolic extracts of leaves and fruit parts of Avocado (*Persea americana*) pear was determined and the result is presented in Fig. 5. The IC_50_ (extract concentration that will inhibit 50% oxidative potential of nitric oxide) revealed that the peel (IC_50_=0.075 mg/mL) had the highest nitric oxide inhibitory potential while the flesh (IC_50_=0.102 mg/mL) had the least.

Characterization of the phenolic extracts with gas chromatography (GC) revealed that the major constituent of the leaves, flesh, seed and peel extract of avocado pear (*Persea americana*) are syringic acid, eugenol, vnillic acid, kaempferol, catechin, epicatechin, ferulic acid, apigenin, naringenin, lupeol, epigallocatechin and epi-gallocatechin-3-O-gallate (Table 3).

**Table 3:** Major phenolics and concentration (mg/100g) of Avocado (*Persea americana*) pear leaves and fruit parts

Phenolics	Leaves Flesh Seed	Peel	Flesh	Seed

Syringic acid	31.65	5.86e^-4^	5.61e^-4^	27.38
Eugenol	21.52	7.22e^-4^	6.48e^-4^	14.53
Vnillic acid	13.33	1.64e^-3^	14.54	10.46
Isoeugenol	12.92	2.07e^-4^	1.89e^-4^	10.73
Guaiacol	12.79	-	-	10.73
Phenol	8.47	-	-	10.13
Kaemferol	8.39	4.48e^-1^	8.68e^-1^	9.07
Catechin	-	4.39	-	-
P-hydroxybenzoic acid	2.51e^-4^	1.37	1.24	7.12e^-4^
Ferulic acid	7.09e^-5^	8.92	11.07	1.54e^-4^
Apigenin	6.06e^-5^	2.13	2.84	2.78e^-4^
Naringenin	7.29e^-4^	3.20	1.86	1.25e^-3^
Epigallocatechin	2.31e^-4^	10.29	34.84	3.06e^-4^
Epicatechin	2.84e^-4^	37.12	46.82	5.53e^-4^
Lupeol	2.61e^-6^	10.89	19.73	2.64e^-6^
Epi-gallocatechin-3-O-gallate	-	13.48	6.03	-

## DISCUSSION

The lowering of postprandial hyperglycemia through the inhibition of key-enzymes linked to type 2 diabetes mellitus (α-amylase and α-glucosidase) is a critical therapeutic strategy used to control/manage type 2 diabetes. α-Glucosidase, after catalyzing the hydrolysis of α, -1, 4-glycosidic bonds to release glucose into the blood stream causes sharp rise in blood glucose level. Delay in the intestinal carbohydrate absorption by α-glucosidase inhibitors slows down this sudden rise in blood glucose level that diabetic patient experience after carbohydrate-rich diet (35). Synthetic oral hypoglycemic agents such as acarbose and miglitol are α-glucosidase inhibitors with severe gastrointestinal side effects. Therefore, the search for natural α-glucosidase inhibitors with little or no side effects could be an attractive approach for the management of this sudden rise in blood glucose level. Inhibitors of these enzymes delay carbohydrate digestion and prolong overall carbohydrate digestion time, causing a reduction in the rate of glucose absorption and consequently reducing the postprandial plasma glucose rise (36).

In this study, the ability of phenolic extracts of *Persea americana* leaves and fruit parts (peel, flesh and seed) to inhibit α-amylase and α-glucosidase activities *in vitro* was investigated and the result presented in Fig. 1 and Fig. 2. The result revealed by the IC_50_ (extract concentration causing 50% enzyme inhibition) values from Table 1 that Peel had the highest significant (*P*<0.05) α-amylase inhibitory activity while the leaf had the highest significant (*P*<0.05) α-glucosidase inhibitory potential. This result is in agreement with a work reported by Jayasri *et al* (37) where aqueous extract of *Coctus pictus D. Don* showed satisfactory inhibitory effect on both α-amylase and α-glucosidase though higher inhibitory effect was shown on α-amylase compared to α-glucosidase activity (37) and the inhibitory effects of *Allium **species*** on α-amylase has also been established (38). This result also falls in line with a recent publication where four seaweeds (*U.lactuca, S. polycystum, G. edulis, and G. corticata*) from the coast of India were studied to find out their possible mechanism of anti-diabetic action. The aqueous extract of *G. edulis* showed sufficient α-glucosidase inhibitory activity while *S. polycystum* revealed better α-amylase inhibitory strength (39).

Sodium nitroprusside is an antihypertensive drug that can also serve as a prooxidant causing cytotoxicity through the release of cyanide and/or nitric oxide (NO) (21). The peel extract showed the highest significant (*P*<0.05) inhibitory potential against malondialdehyde production. The nitric oxide scavenging ability of the four phenolic extracts also revealed that the peel had the highest NO scavenging ability. This result supports a research work where extracts of all the vegetables (tropical green leaf) inhibited malondialdehyde production in rats’ brain with *S. sparganophora* having the highest inhibitory effect while *A.cruentus* had the least (40). This experiment support another where oral supplementation of *Aphanamixis polystachya* bark at a daily dose of 50 and 100 mg body weight for 28 days exhibited significant reduction in hepatic MDA levels when compared with that of the disease control group. All the doses used exhibited statistically significant inhibition of hepatic lipid peroxidation when compared to the control (41). Since the leaf extract also showed the highest NO scavenging ability, the mechanism behind its high inhibitory potential against malondialdehyde production may be attributed to its ability to scavenge nitric oxide (NO) radical produced by sodium nitroprusside.

One of the major antioxidant modes of action is the prevention of the chain initiation step involved in lipid peroxidation by scavenging various reactive species such as free radicals (42). ABTS^*^ scavenging ability of the four extracts of avocado pear (*Persea americana*) also referred to as the total antioxidant capacity was studied using a moderately stable nitrogen centered radical species, ABTS^*^ (32), and this was reported as TEAC (Trolox Equivalent Antioxidant Capacity), as presented in Fig. 4. A higher TEAC value meant that the sample had a stronger antioxidant activity. The result revealed that the leaves extract had the highest ABTS^*^ free radical scavenging potential while the flesh had the least.

Characterization of phenolics of leaves and fruit parts of avocado (*Persea americana*) using gas chromatography (GC) as shown in Table 3 revealed that syringic acid was the main phenolics in leaves and seed while epigallocatechin was the main phenolics in peel and flesh samples.

It has been established by researchers that phenolics can scavenge free radicals, chelate metal catalyst, inhibit oxidases, activate antioxidant enzymes and reduce α-tocopherol radicals (43, 44). One of the mechanisms used by these phenolics is strictly dependent on their ability to stabilize free radicals through the donation of proton (H^+^) (45, 46). These phenolic compounds are involved in the healing process of free radical-mediated diseases including diabetes. It has been suggested that some phenolics may prevent the progressive impairment of pancreatic β-cells function due to oxidative stress and thus reduce the occurrence of type 2 diabetes (47).

## CONCLUSION

Phenolic extracts of avocado Pear (*Persea americana*) leaves and fruit parts (peel, flesh and seed) inhibit α-amylase, α-glucosidase and MDA produced by SNP induced lipid peroxidation in rats’ pancreas. Peel had significant inhibitory power against α-amylase and NO radical. Leaf extract had significant inhibitory potential against α-glucosidase and ABTS^*^free radical. Recent researchers had proved that phenolics from natural resources contain phytochemical constituents that are hypoglycemic in action. Therefore, the strong inhibitory/synergistic potential of the peel and leaves extracts on α-amylase and α-glucosidase respectively may be due to the presence of high concentration of some phenolics revealed by GC which are syringic acid, eugenol, vnillic acid, isoeugenol, guaiacol, phenol, kaemferol, catechin, para-hydroxybenzoic acid, ferulic acid, apigenin, naringenin, epigallocatechin, epicatechin, lupeol and epi-gallocatechin-3-O-gallate. Based on this finding, consumption of avocado pear (*P. americana*) leaves and fruit parts could be a useful means of managing type 2 diabetes and other associated complications arising from oxidative stress. Furthermore, consumption of this fruit should be encouraged in order to benefit from their functional and neutraceutical prowess.
